# Supplementary figures and images for: Transcriptome Sequencing Analysis of circRNA in Skeletal Muscle between Fast- and Slow-Growing Chickens at Embryonic Stages
Source: Animals (Basel). 2022 Nov 16;12(22):3166. doi: 10.3390/ani12223166 (PMC9686870; doi:10.3390/ani12223166)

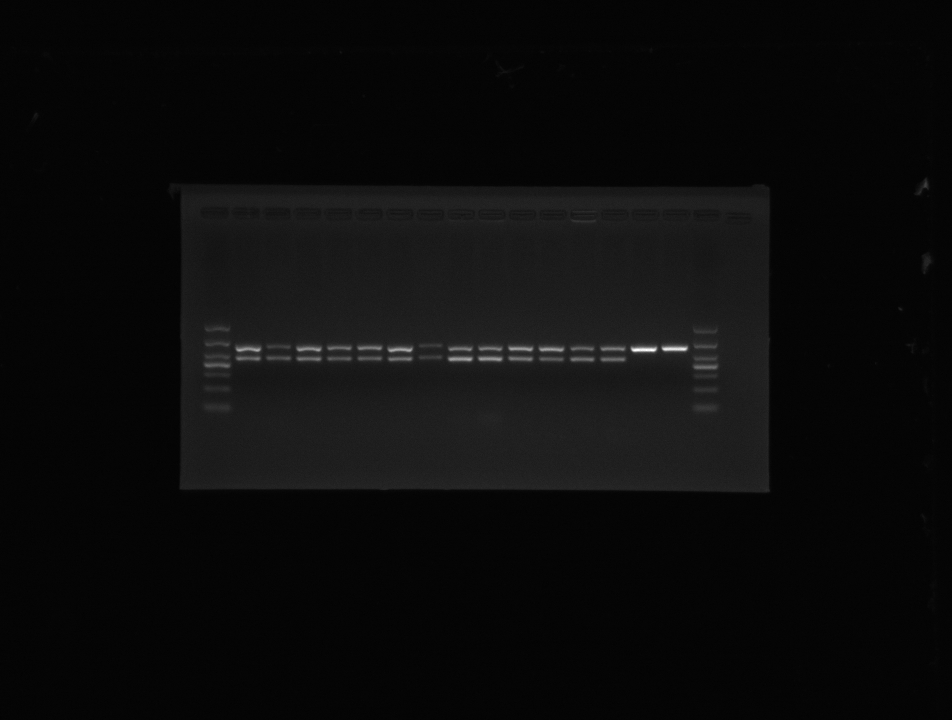

Supplement: Supplementary file 1 [file animals-12-03166-s001.zip › Figure S1 Result of sex identification with CHD1 gene.tif]

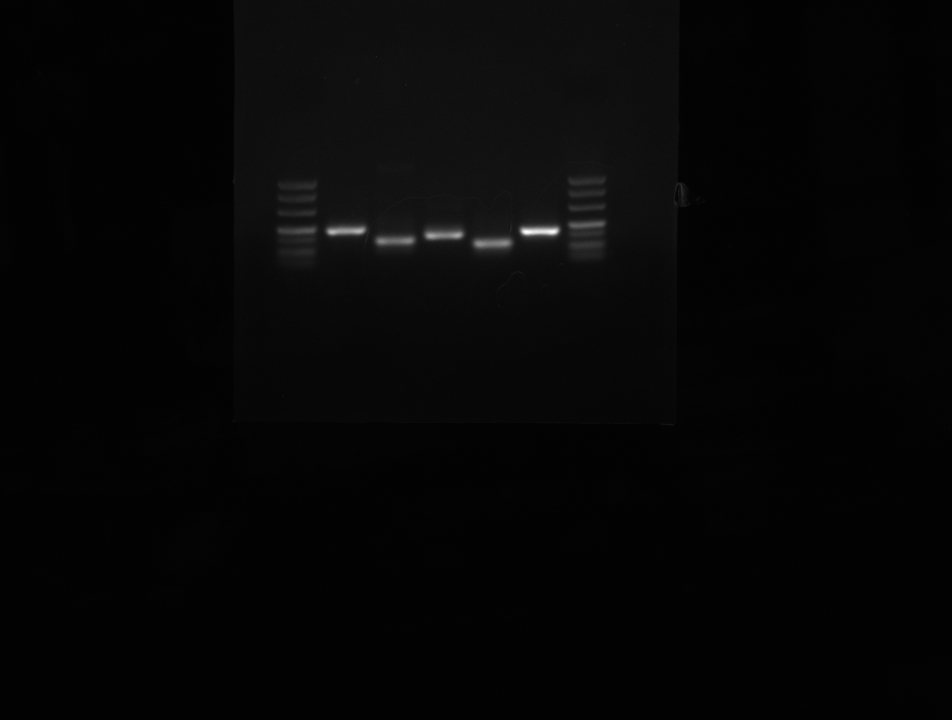

Supplement: Supplementary file 1 [file animals-12-03166-s001.zip › Figure S2 Original agarose gel electrophoresis for PCR products of divergent primers.tif]
